# Sustainable Multi-Network Cationic Cryogels for High-Efficiency Removal of Hazardous Oxyanions from Aqueous Solutions

**DOI:** 10.3390/polym15040885

**Published:** 2023-02-10

**Authors:** Ecaterina Stela Dragan, Doina Humelnicu, Maria Valentina Dinu

**Affiliations:** 1 Department of Functional Polymers, “Petru Poni” Institute of Macromolecular Chemistry, Grigore Ghica Voda Alley 41 A, 700487 Iasi, Romania; 2 Faculty of Chemistry, Alexandru Ioan Cuza University of Iasi, Carol I Bd. 11, 700506 Iasi, Romania

**Keywords:** multi-network cryogels, dichromate ions, phosphate ions, sorption, sustainability

## Abstract

It is still a challenge to develop advanced materials able to simultaneously remove more than one pollutant. Exclusive cationic composite double- and triple-network cryogels, with adequate sustainability in the removal of Cr_2_O_7_^2−^ and H_2_PO_4_^−^ oxyanions, were developed in this work starting from single-network (SN) sponges. Chitosan (CS), as the only polycation originating from renewable resources, and poly(N,N-dimethylaminoethylmethacrylate) (PDMAEMA) and polyethyleneimine (PEI), as synthetic polycations, were employed to construct multi-network cationic composite cryogels. The properties of the composites were tailored by the cross-linking degree of the first network (SN5 and SN20, which means CS with 5 or 20 mole % of glutaraldehyde, respectively) and by the order of the successive networks. FTIR, SEM-EDX, equilibrium water content and compressive tests were used in the exhaustive characterization of these polymeric composites. The sorption performances towards Cr_2_O_7_^2−^ and H_2_PO_4_^−^ anions were evaluated in batch mode. The pseudo-first-order, pseudo-second-order (PSO) and Elovich kinetics models, and the Langmuir, Freundlich and Sips isotherm models were used to interpret the experimental results. The adsorption data were the best fitted by the PSO kinetic model and by the Sips isotherm model, indicating that the sorption mechanism was mainly controlled by chemisorption, irrespective of the structure and number of networks. The maximum sorption capacity for both oxyanions increased with the increase in the number of networks, the highest values being found for the multi-network sponges having SN5 cryogel as the first network. In binary systems, all sorbents preferred Cr_2_O_7_^2−^ ions, the selectivity coefficient being the highest for TN sponges. The high sorption capacity and remarkable reusability, with only a 4–6% drop in the sorption capacity after five sorption–desorption cycles, recommend these composite cryogels in the removal of two of the most dangerous pollutants represented by Cr_2_O_7_^2−^ and H_2_PO_4_^−^.

## 1. Introduction

The presence of highly toxic pollutants, such as heavy metal ions (HMIs) and dyes in aquatic environments, as a consequence of intensive industrialization and of anthropogenic activities, represents the main cause of the deep deterioration of water quality, which threatens public health [1,2,3,4,5]. Among HMIs, Cr(VI) is present in wastewaters in a high concentration as a result of mining activities, electroplating, dyeing, pigment manufacture, leather tanning, pesticides production, textile industries, wood preservation and alloy and steel manufacturing [6]. Cr(VI) compounds present as HCrO_4_^−^, CrO_4_^2−^ and Cr_2_O_7_^2−^ oxyanions (depending on pH and Cr(VI) concentration) are known for their toxicity, high solubility and high diffusivity, which allow them to easily cross the cellular membrane [6]. Cr(VI) is carcinogenic and mutagenic for living organisms and has a corrosive effect on tissues, causing pulmonary congestion and damage of the liver, stomach and kidney [6,7]. Therefore, the World Health Organization has limited the total concentration of chromium in drinking water to 0.05 mg/L. Thus, various methodologies have been employed to decrease the chromium concentration under this limit, such as precipitation, ion exchange, membrane filtration, reverse osmosis and adsorption. Among all known techniques in use for the removal of Cr(VI), adsorption technology is the most widely explored due to its intrinsic advantages, such as lower capital costs, effectiveness, environmental friendliness, due to the absence of further waste materials, and high effectiveness at low concentrations of pollutant compared with other methods [3,6,7]. It is still a challenge to design novel sorbents endowed with enhanced sorption capacity and high stability during utilization. Ion exchangers [8,9,10,11,12] or nitrogen-containing fabrics [13], bearing plenty of quaternary ammonium groups and/or secondary and tertiary amine groups, have demonstrated so far high sorption capacities and reusability in the removal of Cr(VI). Increasing attention has been recently focused on using composite hydrogels in environmental remediation, where they have outcompeted many of the conventional adsorbents by their sorption performances such as fast sorption kinetics and high sorption capacity. Chitosan (CS)-based composites are among the most investigated bio-sorbents with clear performances in the removal of various pollutants [7,14,15,16]. 

Besides Cr(VI), phosphate is a dangerous contaminant, and its concentration in waters requires steady surveillance because concentrations of phosphate higher than 1 ppm in treated wastewater are responsible for the growth of blue-green algae and hyacinth-like plants, which decrease the content of dissolved oxygen, having as the last consequence the death of plants and animals. Various synthetic anion exchangers [17,18,19,20] as well as composite biosorbents, mainly based on cellulose, CS and alginate [17,21,22,23,24,25,26,27,28], have been used in the adsorption of phosphate anions. The selectivity for phosphate ions increased by loading the sorbent with metal ions such as Zr(IV) [19,22,23,24], Fe(III) [24,25] and La(III) [27,28]. Among the synthetic polycations, poly(ethyleneimine) (PEI) [29,30] and poly(N,N-dimethylaminoethyl methacrylate) (PDMAEMA) [31,32] demonstrated high ability in binding various anionic pollutants and heavy metal ions. In our previous investigations on the removal of phosphate anions from aqueous solutions, it has been revealed that the sorption capacity of some composite sponges could be enhanced, increasing the density of amine groups by the fabrication of multi-network cryogels [33,34], following the double network (DN) concept, promoted by Gong et al. [35], and intensively exploited lately by other groups [36,37,38,39]. These results prompted us to extend the study on the sorption performances of multi-network sponges constructed with CS, PEI and PDMAEMA by changing the building order of the networks in DN and triple-network (TN) composites. This approach enlarges the possibilities to diversify the density and distribution of the cationic groups, on the one hand, and the mechanical properties of the composite sponges, on the other hand, and contributes to the increase in the sustainability of these composites in the sorption process of various pollutants. The application of such composites in the removal of Cr(VI) as Cr_2_O_7_^2−^ oxyanions in comparison with phosphate anions is presented for the first time in this work. The composite sponges were characterized by FTIR spectroscopy, SEM-EDX, swelling at equilibrium, and compressive tests. The sorption performances were evaluated as a function of contact time and initial concentration of each oxyanion. To gain information on the interaction mechanism between the composite sorbent and oxyanions, the experimental kinetics and equilibrium data were fitted with some mathematical models. The selectivity of a certain composite cryogel for one of the two oxyanions (Cr_2_O_7_^2−^ and H_2_PO_4_^−^) and the reusability of the selected composites, in connection with their structure, were also thoroughly examined. 

## 2. Materials and Methods

### 2.1. Materials

CS low molecular weight as powder, ethyleneglycol diglycidyl ether (EGDGE) 50%, glutaraldehyde (GA) as aqueous solution with a concentration of 25 wt.%, N,N′-methylenebisacrylamide (BAAm) (99%), ammonium persulfate (APS) (99%) and N,N,N′,N′-tetramethyl ethylenediamine (TEMED) (99%) were supplied from Sigma-Aldrich (Schnelldorf, Germany) and used as received. The deacetylation degree of CS was evaluated by recording its FTIR spectrum in KBr pellets with a Vertex 70 Bruker FTIR spectrometer (Bruker, Ettlingen, Germany). An average value of 85% was obtained from three determinations. The viscometric average molecular weight (M_v_) of CS, assessed according to Ref. [40], was 305 kDa. Two sorts of branched PEI, as aqueous solution with a concentration of 50 wt.%, were used in this work: PEI with average weight molar mass of 1800 g/mole (PEI18) and 25,000 g/mole (PEI25), respectively. N,N-dimethylaminoethyl methacrylate (DMAEMA) (98%), purchased from Sigma-Aldrich, was distilled at 47 °C, at reduced pressure (about 4 mm Hg), and kept at 4 °C. K_2_Cr_2_O_7_ (≥99%) from Sigma-Aldrich (Schnelldorf, Germany) and KH_2_PO_4_ (≥99%) from Merck Chemical Co. (Buchs, Switzerland) were used as sources for oxyanions. 1,5-diphenylcarbazide, (NH_4_)_6_Mo_7_O_24_∙4H_2_O (99%), potassium antimony (III) tartrate hemihydrate (≥99%) were supplied from Sigma-Aldrich (Schnelldorf, Germany) and used as received. Acetic acid, HCl and NaOH were purchased from Chemical Company (Romania) and used as received.

### 2.2. Preparation of Cationic Composite Cryogels

All cryogels prepared and characterized in this work are presented in Table 1. 

Two single-network cryogels consisting of CS cross-linked with GA, with two concentrations, i.e., 5 mole % and 20 mole %, the sample codes being SN5 and SN20, respectively, were prepared first following the protocol presented in the previous paper [34]. For the fabrication of DN and TN composites having SN20 as the first network, the following steps were taken. To obtain SN20 sponges, 2.44 mL aqueous solution of GA, with a concentration of 2.5 wt.%, was dropped under vigorous magnetic stirring on 30 g aqueous solution of 2 wt.% CS in 2 vol % CH_3_COOH in an ice bath. The stirring went on for 45 min after the addition of GA and then the reaction mixture was loaded in syringes of 10 mL, sealed with parafilm and transferred to a freezer at −18 °C for 24 h. The monoliths were then kept for 30 min at room temperature, cut at about 10 mm length and immersed in distilled water for about 72 h. The SN monoliths were freeze-dried in a Martin Christ, ALPHA 1-2LD device, for 48 h at −57 °C and 0.045 mbar. The fragments of SN cryogels were loaded at saturation with (1) a monomer mixture consisting of DMAEMA (10 wt.%), BAAm (5 moles BAAm: 100 moles of DMAEMA), TEMED and APS (2 wt.% to monomer and cross-linker) to prepare DN20.1 (Table 1); (2) aqueous solution of PEI25 containing 5 mole % of EGDGE thus obtaining the composite DN20.2 (Table 1); and (3) aqueous solution of PEI18 (C = 15 wt.%) containing 5 mole % of EGDGE, thus obtaining DN20.3 (Table 1). To prepare the third networks, namely TN20.1, TN20.2 and TN20.3 composite cryogels, the DN20.1, DN20.2 and DN20.3 cryogels, respectively, were swollen at saturation in an aqueous solution of PEI18 (10 wt.%) containing EGDGE in a molar ratio of 5 moles EGDGE: 100 moles PEI (Table 1). A monomer mixture consisting of DMAEMA (10 wt.%), BAAm (5 moles BAAm: 100 moles of DMAEMA) TEMED and APS (2 wt.% to monomer and cross-linker) was adsorbed into DN5 cryogel to obtain TN5.2 composite (Table 1). The formation of the third network was conducted in a cryostat at −18 °C. After 24 h, the TN cryogels were thawed at room temperature (RT) and intensively washed with distilled water for at least 72 h to leach out all unreacted compounds. The TN composite cryogels were finally dried by lyophilization. 

### 2.3. Characterization of Cationic Composite Cryogels

The FTIR spectra in the 4000–400 cm^−1^ range were recorded on a Bruker Vertex FTIR spectrometer by the KBr pellet technique. An amount of about 5 mg of sample was placed in each pellet. All FTIR spectra were processed by ACD/Spec Viewer 5.04 Software. To evaluate the internal morphology and the elemental composition of the cryogels, a Quanta 200 environmental scanning electron microscope (ESEM) (FEI Company, Hillsboro, Oregon, USA) type Quanta 200) coupled with an energy-dispersive X-ray (EDX) detector was used. The ImageJ 1.48v software was employed to determine the average diameter of pores and the thickness of the pore walls of the cryogels. In this regard, three independent SEM micrographs were utilized, and the values were reported as the average of at least 15 pores ± standard deviations [41].

Swelling ratio (*SR*) was evaluated by immersing samples of composite cryogels (about 0.01 g) in 20 mL distilled water, up to the equilibrium of swelling. The samples were weighed after the removal of water in excess, and the *SR* values was calculated with Equation (1) [42]:

(1) $$SR=\frac{W_{eq}}{W_{d}}$$

where *W_eq_* represents the mass of swollen gel at equilibrium, and *W_d_* is the mass of cryogel in dry state.

Equilibrium water content (*EWC*) was evaluated by Equation (2) [43]:

(2) $$EWC=\left[\left(W_{eq}-W_{d}\right)/W_{d}\right]\times 100$$

where *W_eq_* and *W_d_* have the same meaning as in Equation (1).

To assess the mechanical behavior of SN, DN and TN cryogels, uniaxial compression measurements were performed with a Shimadzu Testing Machine (EZ-LX/EZ-SX Series, Kyoto, Japan) on equilibrium swollen samples. All cryogels were cut as monoliths with a diameter of about 12–14 mm and a height of 4–6 mm. Before each compression test, an initial force of 0.1 N was applied to ensure the complete contact between the surface of cryogels and the plates of the testing equipment. The stress–strain profiles were recorded using a cross-head speed of 1 mm min^−1^ and a force of 50 N. The compressive stress (*σ*, kPa), strain (*ε*) and the elastic moduli (*G*, kPa) were calculated according to the previously published protocols for other porous-based materials [34,44,45,46].

### 2.4. Sorption of Oxyanions

Batch sorption of oxyanions onto the SN, DN and TN cryogels was carried out in a temperature-controlled shaker (GFL 1083, Gemini BV, Steinfurt, Germany), vibrated at ~160 rpm, using 0.0075 g of sorbent and 10 mL of oxyanion solution. The pH of the solutions was adjusted with 0.1 M HCl or 0.1 M NaOH. After shaking for a certain time, the sorbent was separated by filtering through a 0.45 μm membrane filter. The evaluation of the concentration of Cr(VI) was performed by the 1,5-diphenylcarbazide method, in acidic medium, using a UV–Vis spectrophotometer (Hitachi U-2001, λ = 540 nm) [8,47]. The concentration of phosphate ions in aqueous solution was determined by the colorimetric method in the presence of ammonium molibdatum and potassium antimonyl tartrate using ascorbic acid as a reduction agent (λ = 888 nm), with a UV–Vis Hitachi U-2001 spectrophotometer (Hitachinaka, Ibaraki 312-8504, Japan) [48]. The mean of three readings for each sample was used for the calculation of the sorption results. The adsorption capacity at a certain contact time, *q_t_* (mg/g), was calculated with Equation (3):

(3) $$q_{t}=\frac{(C_{o}-C_{t})V}{m}$$

where *C_o_*—initial concentration of pollutant (mg/L), *C_t_*—concentration of pollutant in the aqueous solution at a certain duration (mg/L), *V*—volume of the aqueous solution (L), *m*—mass of sorbent (g).

The removal efficiency (*RE*) was calculated with Equation (4):

(4) $$RE=\frac{C_{o}-C_{e}}{C_{o}}\times 100$$

where *C_o_* has the same meaning as in Equation (4) and *C_e_* is the concentration of pollutant in the aqueous solution at equilibrium.

The evaluation of kinetics and isotherm parameters was performed by a non-linear regression method using two error functions to assess the level of fit: the correlation coefficient of determination (*R^2^*) and the non-linear Chi-square (*χ^2^*) test, calculated by Equation (5):

(5) $$\chi^{2\;}=\sum \frac{{\left(q_{e,exp}-q_{e,cal}\right)}^{2}\;}{q_{e,cal}}$$

where *q_e,exp_* and *q_e,cal_* represent the experimental data (mg/g) and the data calculated by models (mg/g), respectively.

Sorption of oxyanions in di-component systems was performed in the following conditions: V_sol_ = 10 mL; sorbent dose 0.0075 g; *C_o_* = 100 mg/L (for each oxyanion); 25 °C; contact time 4 h. The selectivity coefficient for the sorption of oxyanions was evaluated with Equation (6):

(6) $$k_{H_{2}PO_{4}^{-}}^{Cr_{2}O_{7}^{2-}}=\frac{K_{dCr_{2}O_{7}^{2-}}}{K_{dH_{2}PO_{4}^{-}}}$$

where $K_{dCr_{2}O_{7}^{2-}}$ and $K_{dH_{2}PO_{4}^{-}}$ represent the distribution constants of Cr_2_O_7_^2−^ and H_2_PO_4_^−^ oxyanions, respectively. 

## 3. Results

### 3.1. Synthesis and Characterization of Multi-Network Cationic Composite Cryogels

#### 3.1.1. Synthesis

**Figure 1:**
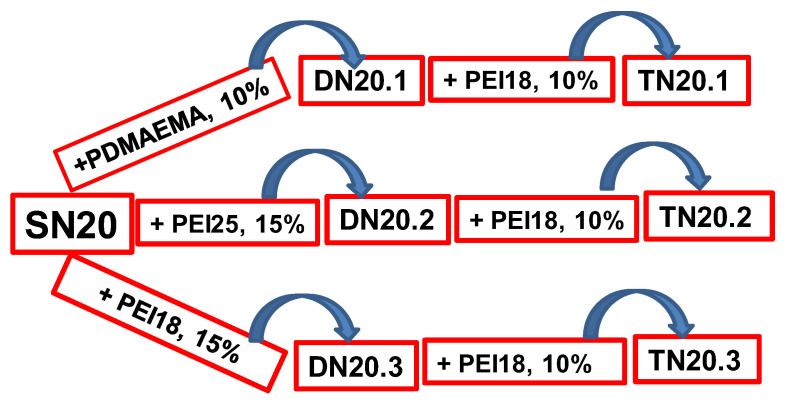
Synthesis strategy of DN and TN composite cryogels starting from the single network of CS cross-linked with 20 mole% GA.

The principle which is based on the fabrication of multi-network architectures has already been described [33,34,35,36]. This strategy is accessible and extremely versatile given the possibility to increase, in a controlled manner, the density of a certain category of functional groups. Figure 1 summarizes the strategy used in this work to spread out the structure of the DN and TN composite cryogels starting from the single-network SN20. 

As can be seen in Figure 1, the second network was generated either by cross-linking polymerization of DMAEMA at a concentration of 10 wt.%, thus obtaining the composite DN20.1, or by the cross-linking with EGDGE of PEI having two molar masses (PEI25 and PEI18), thus obtaining the composites DN20.2 and DN20.3, respectively. The third network was the same for all TN cryogels, i.e., PEI18 (10 wt.%) (Table 1, Figure 1). In all cases, PEI was cross-linked with 5 mole% EGDGE. 

#### 3.1.2. Characterization

**Figure 2:**
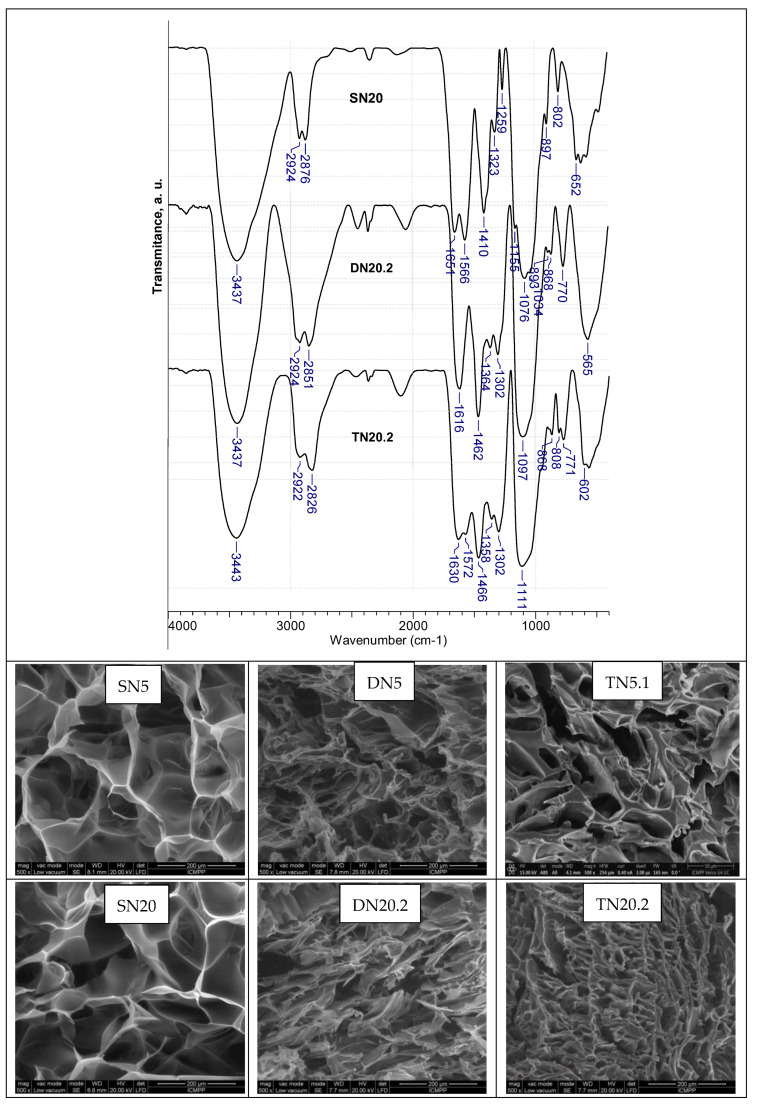
FTIR spectra of SN20, DN20.2 and TN20.2 (Table 1) (up) and SEM images of SN5, DN5, TN5.1, SN20, DN20.2 and TN20.2 (Table 1) (down) with scale bar of 200 µm and magnification of 500×.

Figure 2 presents the FTIR spectra of the series SN20 (CS), DN20.2 (CS/PEI25) and TN20.2 (CS/PEI25/PEI18) (from Figure 1). As expected, the main bands of CS are visible in the spectrum of SN20 cryogel, as follows: 2924 and 2876 cm^−1^, attributed to C-H and -CH_2_ stretching vibrations in CS; 1651 cm^−1^, assigned to the stretching vibration of C=O bond in the secondary amide bond and to the Schiff base; 1566 and 1410 cm^−1^, attributed to the –NH bending vibration in amide II, and to C-H and -CH_2_ stretching, respectively; 1323 cm^−1^, assigned to N-H stretching in amide (amide III); 1259 cm^−1^, assigned to the C-N stretching modes; the large band at 1076 cm^−1^ indicates the C-O-C anti-symmetric stretching in CS [49,50,51]. By the construction of the second network (PEI25), significant changes in the position of the main bands are obvious in the spectrum of the DN20.2 composite. The sharp band located at 1462 cm^−1^ supports the presence of PEI, while the bands at 1364 and 1302 cm^−1^ indicate CH_3_ symmetrical deformation mode and the amide III [49]; the bands at 1097 and 943 cm^−1^ were assigned to the C-O-C anti-symmetric stretching, and the wagging of the saccharide structure of CS. The construction of the third network (PEI18) in TN20.2 did not lead to dramatic changes in the spectrum, just small blue or red shifts; for example, the band at 1566 cm^−1^ in SN20 was blue-shifted at 1572 cm^−1^ in TN20.2, the band at 1462 cm^−1^ was blue-shifted to 1466 cm^−1^, the band at 1364 cm^-1^ in DN20.2 was shifted at 1358 cm^-1^ and the band at 943 cm^−1^ appears as a shoulder after the construction of the third network, while the band at 897 cm^−1^ in SN20, assigned to the wagging of saccharide structure, was red-shifted at 893 cm^−1^ in DN20.2 and at 868 cm^−1^ in TN20.2.

The FTIR spectra of SN5, DN5 and TN5.2 sponges are compared in Figure S1. The bands located at 2924 and 2677 cm^−1^ in SN5, at 2924 and 2822 cm^−1^ in DN5, and at 2926 and 2822 cm^−1^ in TN5.2 were attributed to C-H and -CH_2_ stretching vibrations; the bands situated at 1632, 1651 and 1631 cm^−1^ in SN5, DN5 and TN5.2, respectively, were assigned to the stretching vibration of C=O bond in the secondary amide bond and to the Schiff base. The large bands located at 1076 cm^−1^ in SN5, 1115 cm^−1^ in DN5 and 1159 cm^−1^ in TN5.2 indicate the C-O-C anti-symmetric stretching in CS [49,50,51]. The sharp bands located at 1442 cm^−1^ in SN5, 1462 cm^−1^ in DN5 and 1451 cm^−1^ in TN5.2 were assigned to methylene C-H bending vibrations [51]. The presence of PDMAEMA as the third network is supported by the band at 1731 cm^−1^ attributed to the C-O bond in the ester group and the strong band at 1451 cm^−1^. Figure S2 presents the FTIR spectra of DN20.3 and TN20.3 composite sponges. As can be seen, these spectra are similar because the second and the third networks were constituted of PEI18 cross-linked with EGDGE. 

SEM images in Figure 2 support the morphological changes which occurred during the successive construction of the networks. The pore sizes and the wall thickness become smaller and bigger, respectively, after the addition of the second and third networks, both for SN5 and SN20 as the first network. It is obvious that the compactness of the composites was higher when the first network was SN20, the theoretical cross-linking degree being four times higher compared with that of SN5. The size of pores and the wall thickness for some representative composite cryogels are presented in Table S1 and show the dramatic decrease in the pore sizes by the construction of the second network (DN5 compared with SN5 and DN20.2 compared with SN20). The pore size and pore wall thickness further decreased and increased, respectively, by the construction of the third network (Table S1). 

The elemental analyses obtained from EDX spectra, and given in Table S2, support the structure of the composites. As can be seen, the value of nitrogen content in the SN20 cryogel is lower than in the SN5 cryogel because a higher content of GA had as a consequence increasing carbon content and decrease of nitrogen and oxygen content. The construction of the PEI network led to the increase in carbon and nitrogen content and to the decrease in oxygen content (DN5 and DN20.2). The strong difference between the values of elements in the case of the composites TN5.1 and TN5.2 is attributed to the third network, which was PEI in the first case and PDMAEMA for TN5.2. The difference between the element content in the case of DN20.2 and TN20.2 was not so significant because the second and the third networks were constituted of PEI.

The values of *SR* and *EWC*, presented in Table S3, clearly indicate the differences between SN5 and SN20 caused by the differences in cross-linking degrees. Thus, for SN5, the values of *SR* and *EWC* were 64.69% and 98.45%, respectively, while for SN20, the values of these characteristics were 45.25% and 97.79%. The values of *SR* and *EWC* dramatically decreased with the construction of the second network (DN5, DN20.1, DN20.2 and DN20.3), while the decrease was less significant when the third network was added (TN5.1, TN5.2, TN20.1, TN20.2 and TN20.3).

**Figure 3:**
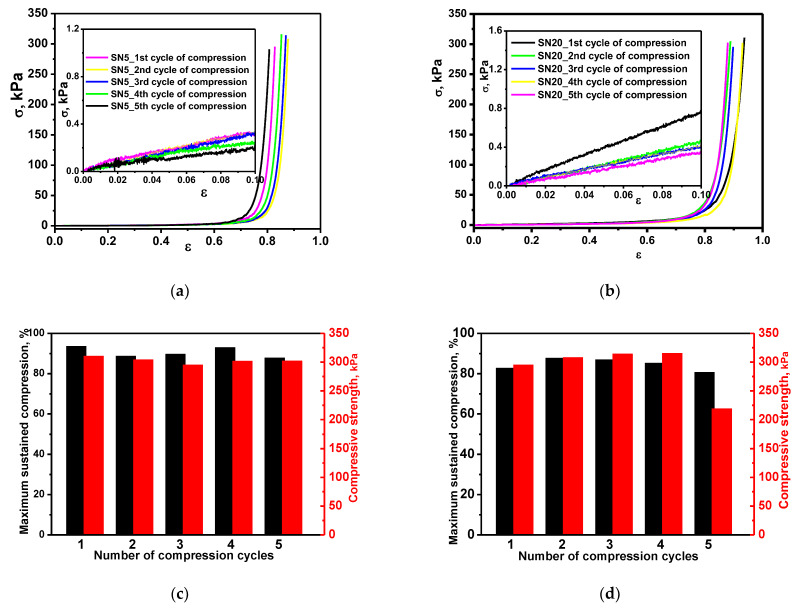
The mechanical properties of single-network cryogels as a function of number of compression cycles. Stress–strain profiles of SN5 (**a**) and SN20 (**b**) cryogels (the insets of (**a**,**b**) present the linear dependence of stress–strain curves used for the calculation of compression elastic moduli). Maximum sustained compression (black column bars) and compressive strength (red column bars) for SN5 (**c**) and SN20 (**d**) cryogels.

**Figure 4:**
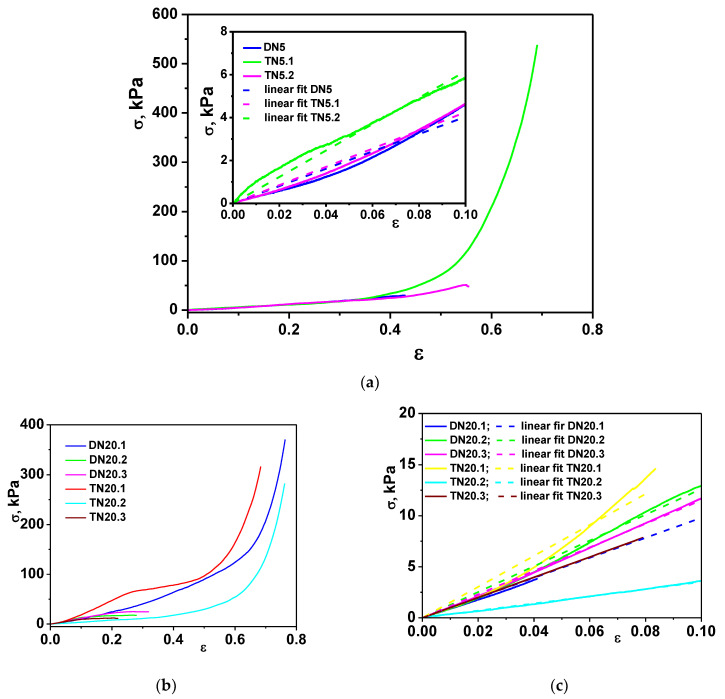
The mechanical properties of DN and TN network cryogels. Stress–strain profiles of DN and TN obtained starting from SN5 (**a**) and SN20 (**b**) cryogels (the inset of (**a**) presents the linear dependence of stress–strain curves used for the calculation of compression elastic moduli of DN5 and TN5 cryogels). (**c**) Linear dependence of stress–strain curves from which the compression elastic moduli of the DN20 and TN20 cryogels were determined.

Uniaxial stress–strain compression tests were first performed to evaluate the mechanical strength of the SN cryogels, namely SN5 and SN20. The stress–strain curves presented in Figure 3a,b show typical elastic behavior characteristic of cryogels, with sustained compression values beyond 70% and without any deformation or failure of the gel networks. Moreover, after the removal of the load, the SN5 and SN20 cryogels reabsorbed the water released during compression and regained their original shape. Consequently, in order to further evaluate their resilience and robustness, the dynamic stress–strain behavior of these cryogels was carried out for five successive loading cycles at 95% maximum strain. The results in Figure 3c,d and Table S4 (Supplementary Materials) demonstrate the remarkable mechanical stability of the SN5 and SN20 cryogels upon successive compression experiments; the values of the maximum sustained compression, the compressive strength and the compressive moduli remained almost unchanged after five successive cycles of compression. It should be pointed out that SN5 and SN20 cryogels exhibited unique mechanical features (high elasticity, non-brittleness, shape recovery), which support their further use as matrices/scaffolds for construction of multi-network composite cryogels. By employing PEI18 and EGDGE to prepare the second network, stiffer DN cryogels were obtained, which can sustain 53% (sample DN5) and 31.18% (sample DN20.3) compression before fracture, respectively (Figure 4 and Table 2). The crack development in DN cryogels at low strain can be associated with the drastically decreased values of the swelling ratio of the DN cryogels in comparison with those of SN cryogels (see Table S4, Supplementary Materials).

However, the compressive moduli of DN cryogels, calculated as the gradient of the initial linear portion in the stress–strain curve (Figure 4c), increased with the addition of the second network. For instance, DN5 and DN20.3 cryogels exhibited an elastic modulus about five and thirty times higher than that of the starting networks (SN5 and SN20), respectively. Thus, increasing the polymer content led to more dense and rigid networks. Moreover, the construction of the third network based on PEI18 or PDMAEMA matrices led to a further improvement of the elastic modulus of the composite cryogels (samples TN5.1 and TN5.2; Table 2). Furthermore, the TN5.1 cryogels remained mechanically stable and sustained 69.02% compression at a compressive nominal stress of 538.2 kPa (Figure 4a and Table 2). Thus, the use of PEI18 to prepare the third network increased the flexibility of the TN5.1 network and hampered its failure. On the other hand, when SN20 cryogels, having a higher cross-linking density (GA concentration 20 mole %), were used as starting networks for the construction of the TN networks (sample TN20.3), the mechanical properties were completely changed. A cross-linker content of 20% in the first network (TN20.3) induced an enhancement in the elastic modulus compared to a cross-linker content of 5% (TN5.1), but the fracture of the TN20.3 networks occurred at lower compression ratio (21.91%), while the TN5.1 cryogels were robust without deformation or fracture at higher strain.

Additionally, the uniaxial compression data proved that the PDMAEMA chains had a beneficial effect on the maximum sustained compression and compressive strength of DN cryogels (Figure 4 and Table 2). DN20.1 cryogels demonstrated mechanical stability and sustained 76.3% compression at a compressive nominal stress of 370.49 kPa, whereas DN20.2 and DN20.3 cryogels were broken at about 13.30% and 31.18% compression and compressive nominal stress of 14.45 kPa and 24.75, respectively (Figure 4b and Table 2). Nevertheless, the presence of a third network based on PEI18 determined a significant reinforcement of the TN20.2 cryogel network, which sustained 76.15% compression, at a compressive nominal stress of 282.35 kPa. In addition, the remarkable mechanical stability of TN5.1 and TN20.2 can be also associated with the well-interconnected networks of small pores observed for these cryogels (see SEM micrographs, Figure 2). An improvement of the mechanical strength with the decrease in pore sizes has also been previously reported for other porous hydrogels [34,52,53]. Finally, it should be emphasized that the mechanical properties of SN, DN and TN cryogels can be modulated by controlling (i) the cross-linker ratio of the first network, (ii) the nature and molar mass of the polycation used to prepare the second and third networks and (iii) the order of the network construction. 

### 3.2. Sorption Performances

#### 3.2.1. Sorption Kinetics

**Figure 5:**
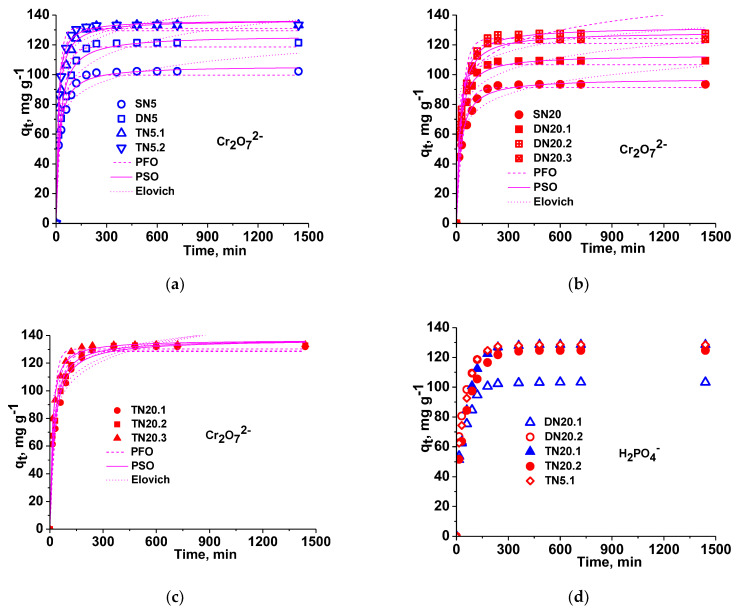
Comparative sorption kinetics of Cr_2_O_7_^2−^ anions onto SN, DN and TN composite sorbents (**a**–**c**). Sorption kinetics of H_2_PO_4_^−^ onto DN and TN composite sorbents (**d**); sorbent dose = 7.5 mg, Vsol = 10 mL, pH = 3 for SN and DN, and pH = 4 for TN; temp = 23 °C; C_in_ = 100 mg/L.

**Figure 6:**
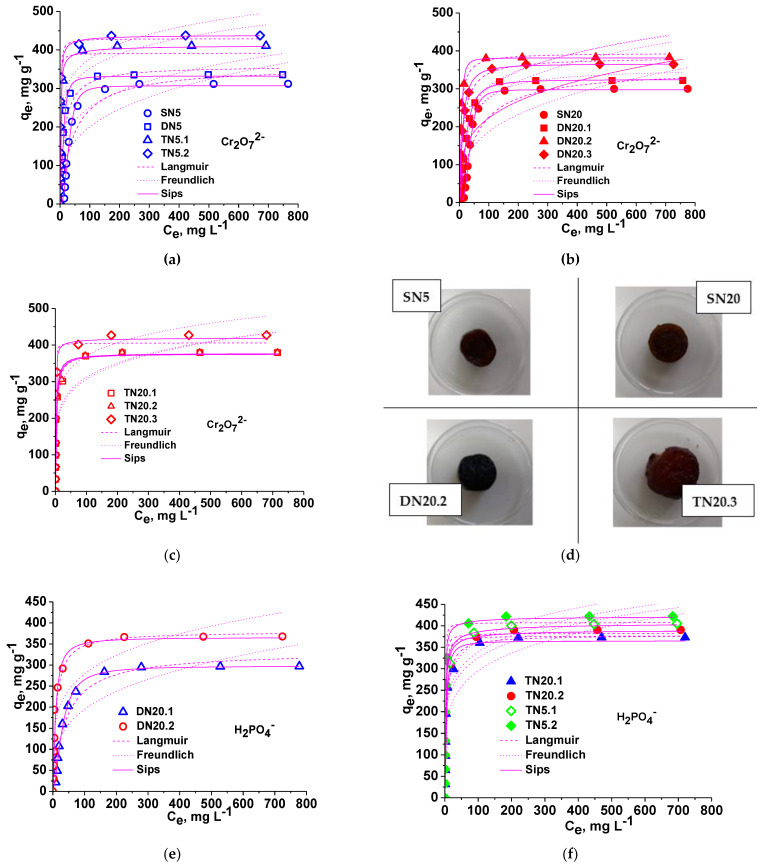
(**a**–**c**) Sorption isotherms of Cr_2_O_7_^2−^ anions onto SN, DN and TN cryogels; sorbent dose = 0.0075 mg; V_sol_ = 10 mL; temp. = 23 °C; pH = 3 for SN and DN, and pH = 4 for TN; contact time = 24 h. (**d**) Optical images of some cryogels after their loading with Cr(VI). Sorption isotherms toward H_2_PO_4_^−^ onto DN (**e**) and TN (**f**) composite cryogels.

Sorption kinetics data obtained for the sorption of Cr_2_O_7_^2−^ oxyanions onto SN, DN and TN cryogels were plotted in Figure 5a–c. As Figure 6a shows, the values of *q_t_* at equilibrium increased with the increase in the number of cationic networks. For the same CS network, the values of sorption at equilibrium increased in the order SN < DN < TN. Moreover, it is obvious that the sorption at equilibrium was higher in the case of both TN cryogels, having SN5 as the first network, the difference consisting of the faster reaching of equilibrium in the case of TN5.2 composite, i.e., when the last network was PDMAEMA. These results are assigned to the higher number of cationic groups available for the electrostatic attraction of Cr_2_O_7_^2−^ oxyanions, in the case of a lower cross-linking degree. Figure 6d shows that the sorption of H_2_PO_4_^−^ was also high and fast, the values being comparable with those of Cr_2_O_7_^2−^.

By the non-linear fitting of the experimental kinetic data obtained for the sorption of Cr_2_O_7_^2−^ anions, the values of the kinetic parameters were obtained. 

As can be seen in Tables S5–S7, the calculated values of q_e_ better agreed with the experimental data in the case of the PSO kinetic model. The values of *R*^2^ and *χ*^2^ are the highest and the lowest, respectively, and show a high level of fit for this model. The PSO kinetic model is considered as the rate-limiting step for chemisorption as the most probable mechanism of sorption, which involves valence forces through sharing or exchange of electrons between all sorbents investigated in this work and Cr(VI) species [9,10,12,15,16,21]. The values of *R*^2^ found when the kinetic data were fitted with the Elovich model, which are in the range of 0.94–0.95, also support chemisorption as a possible mechanism of sorption. 

The sorption kinetics of H_2_PO_4_^−^ anions onto the novel multi-network cationic sorbents (DN20.1, DN20.2, TN20.1, TN20.2 and TN5.1; Table S8) confirm the previous reported results [34]. Chemisorption of this oxyanion is also supported by the highest values of *R^2^* and the lowest values of *χ^2^* in the case of PSO model compared with PFO model. 

#### 3.2.2. Isotherms

Sorption capacity at equilibrium was investigated to identify the sorbents with the best performances in the removal of Cr_2_O_7_^2−^ oxyanions. The experimental data and the fitting of three isotherm models (Langmuir, Freundlich and Sips) are presented in Figure 6a–c. As can be seen in Figure 6, the affinity of the cryogels for Cr_2_O_7_^2−^ anion was high in the case of composites derived from SN5 cryogel, increasing with the increase in the network number (TN5.1 compared with DN5). The sorption capacity was the highest when PDMAEMA was the third network (TN5.2).

The isotherm parameters for the sorption of Cr_2_O_7_^2−^ anions are presented in Table 3, Table 4 and Table 5. As Table 3 shows, the experimental isotherms for the sorption of Cr_2_O_7_^2−^ anions onto SN5 and derived composite cryogels were best fitted by the Sips isotherm model, the calculated values of *q_m_* being close to the experimental values for all sorbents. The values of *R^2^* and *χ^2^* were the highest and lowest, respectively, for this isotherm, thus supporting this isotherm as the most suitable to fit the experimental data. The highest value of *q_m_* was found in the case of the composite cryogel TN5.2, which has three networks, the last one being PDMAEMA.

The sorption data included in Table 4 show how the sorption capacity of Cr_2_O_7_^2−^ anions is influenced by the structure of the second network when the first network was SN20 cryogel. As can be seen, the lowest sorption capacity was found in the case of SN20 (300 mg Cr_2_O_7_^2−^/g sorbent), even lower than that of SN5 (312 mg Cr_2_O_7_^2−^/g sorbent), and the highest in the case of the composite DN20.2, i.e., when the second network was PEI25 (383 mg Cr_2_O_7_^2−^/g sorbent). The difference between the sorption capacity of DN20.2 and DN20.3 (364.6 mg Cr_2_O_7_^2−^/g sorbent) is not so high and could be associated with the difference in the molar mass of PEI used as the third network. As can be observed, the experimental data were also the best fitted by the Sips isotherm model.

Table 5 presents the isotherm parameters corresponding to the three TN composites derived from single-network SN20, the last network being the same for all composites (PEI18, 10%, Table 1). The highest sorption capacity was found in the case of composite TN20.3, while the sorption capacity of TN20.1 and TN20.2 was almost the same (379 and 380 mg Cr_2_O_7_^2−^/g sorbent, respectively). 

By comparing the values of the sorption capacity of Cr_2_O_7_^2−^ anions onto the single-network and composite cryogels we can conclude that the highest sorption capacity could be developed in the case of the composite cryogel TN5.2, i.e., when the first network was SN5, and the last network was PDMAEMA (Table 3). Figure 6d presents the optical images of SN5, SN20, DN20.2 and TN20.3 after their loading with Cr_2_O_7_^2−^ anions, at equilibrium. As can be observed, all sponges display a uniform intensity of color, which supports a uniform distribution of oxyanions inside the sorbents, a qualitative test which indicates the high affinity of these cryogels for Cr_2_O_7_^2−^ anions. 

The experimental isotherms for the sorption of H_2_PO_4_^−^ ions onto some novel DN and TN composite sponges are presented in Figure 6e (DN20.1 and DN20.2) and Figure 6f (TN20.1, TN20.2, TN5.1 and TN 5.2), respectively, and the isotherm parameters obtained by fitting the Langmuir, Freundlich and Sips isotherm models are given in Table 6. As shown in Table 6, the structure of the second network induced a significant difference between the values of the sorption capacity toward H_2_PO_4_^−^ ions. Thus, the values of *q_m_* found in the case of DN20.2 as a sorbent (the second network being PEI25, Table 1) were much higher than those found for DN20.1 (with PDMAEMA as the second network), both experimental values and calculated by fitting Langmuir and Sips isotherm models. This difference could be attributed to the higher number of amino groups in PEI than in PDMAEMA. A consistent increase in the sorption capacity occurred when the third network was constructed (PEI18 for both TN20.1 and TN20.2). It can be also observed that the TN5.1 and TN5.2 sponges, having as the first matrix SN5 cryogel, display the highest values of *q_m_*, in agreement with the values obtained when the adsorption of Cr_2_O_7_^2−^ anions was investigated (Table 3). 

The selectivity of some sorbents for Cr_2_O_7_^2−^ and H_2_PO_4_^−^ anions, in binary mixtures, was investigated, the results being presented in Table S9. It is obvious that all sorbents prefer Cr_2_O_7_^2−^ anions, with the values of the selectivity coefficient, evaluated with Equation (6), increasing from 1.19 (SN5) to 2.77 (TN5.2) and from 1.14 (SN20) to 2.51 (TN20.3). 

The maximum sorption capacity of the best multi-network sponges, prepared in this work, against Cr_2_O_7_^2−^ and H_2_PO_4_^−^ anions is compared in Table S10 with data from the literature. As can be seen from Table S10, the uptake of the two oxyanions on the novel composite cryogels was comparable or even higher than the maximum sorption capacity of other sorbents. 

#### 3.2.3. Reusability

**Figure 7:**
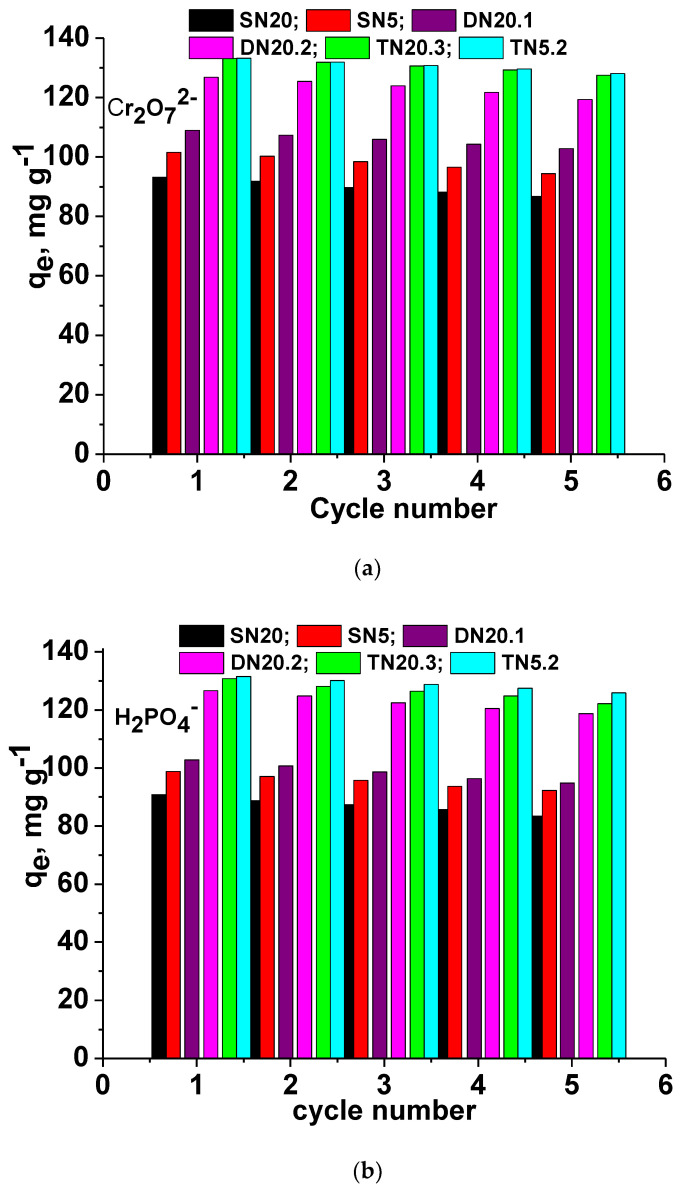
Sorption capacity of Cr_2_O_7_^2−^ (**a**) and H_2_PO_4_^—^ (**b**) oxyanions onto SN, DN and TN cryogels as a function of the adsorption/desorption cycle number.

Sustainability of the novel composite sorbents is an essential feature for their applicability. Therefore, the level of preservation of the sorption capacity of the two SN sponges (SN5 and SN20), two DN composites (DN20.1 and DN20.2) and two TN composites (TN20.3 and TN5.2) toward Cr_2_O_7_^2−^ and H_2_PO_4_^−^ anions is presented in Figure 7a,b, respectively. Comparing the sorption capacity toward Cr_2_O_7_^2−^ anions in the first cycle with that found after the fifth cycle, the loss was around 7%, 6% and 4% in the case of SN, DN and TN composite cryogels, respectively. The loss of the sorption capacity was slightly higher when H_2_PO_4_^−^ anions were adsorbed, the decrease being 8.14%, 6.62%, 7.75%, 6.21%, 6.59% and 4.3% when the sorbent was SN20, SN5, DN20.1, DN20.3, TN20.3 and TN5.2, respectively. The preservation of the sorption capacity for both Cr_2_O_7_^2−^ and H_2_PO_4_^−^ anions of the cationic composite sponges investigated in this work supports their potential as sorbents for the removal of two of the most harmful pollutants present in insufficiently treated wastewaters.

The increase in the sustainability with the increase in the network numbers is obvious for both oxyanions (TN compared with DN sponges).

## 4. Conclusions

Exclusive cationic composite DN and TN sponges, with adequate sustainability in the removal of Cr_2_O_7_^2−^ and H_2_PO_4_^−^ oxyanions, were developed in this work, starting from self-healing SN sponges based on CS. PEI and PDMAEMA were used as synthetic polycations in the construction of the second and third networks. The properties of the composite sponges were adjusted by the cross-linking degree of the first network (5 or 20 mole% GA) and by the order of the successive networks of PEI and PDMAEMA. The sorption data were best fitted by the PSO kinetic model, indicating that the sorption mechanism was mainly controlled by chemisorption, irrespective of the structure and number of networks, while the experimental isotherms were best described by the Sips isotherm model. The benefits of the multi-network cationic sponges in the sorption of hazardous oxyanions include the monotonous increase in the sorption capacity of the composites with the increase in the number of networks. Starting from SN20 as the first network, the maximum sorption capacity calculated by the Sips isotherm model increased from 298 mg Cr_2_O_7_^2−^/g SN20 to 366 mg Cr_2_O_7_^2−^/g DN20.3, and to 420 mg Cr_2_O_7_^2−^/g TN20.3. When SN5 was the first network, the increase in the sorption capacity was in the following order: 307 mg Cr_2_O_7_^2−^/g SN5, 332 mg Cr_2_O_7_^2−^/g DN5 and 439 mg Cr_2_O_7_^2−^/g TN5.2. The selectivity coefficient for Cr_2_O_7_^2−^ anions, in binary systems, increased with the increase in the network number for both series of cationic composite sponges. This new strategy may provide a universal approach to fabricate multi-network cryogels, based on natural polysaccharides as the first network, and two synthetic polycations (PEI and PDMAEMA) endowed with superior mechanical properties, a high level of recyclability and versatile functions.

## Figures and Tables

**Table 1:** Double-network (DN) and triple-network (TN) cryogels prepared and used as sorbents for the removal of Cr_2_O_7_^2−^ and H_2_PO_4_^−^ oxyanions.

Cryogel Name	1st Network		2nd Network		3rd Network	
	Name *	GA, Mole %	Name	Conc. wt.%	Name	Conc. wt.%
SN5	CS	5	-	-	-	-
DN5	CS	5	PEI18	15	-	-
TN5.1	CS	5	PEI18	15	PEI18	10
TN5.2	CS	5	PEI18	15	PDMAEMA	10
SN20	CS	20	-	-	-	-
DN20.1	CS	20	PDMAEMA	10	-	-
DN20.2	CS	20	PEI25	15	-	-
DN20.3	CS	20	PEI18	15	-	-
TN20.1	CS	20	PDMAEMA	10	PEI18	10
TN20.2	CS	20	PEI25	15	PEI18	10
TN20.3	CS	20	PEI18	15	PEI18	10

* CS concentration was 2 wt.% in all syntheses.

**Table 2:** Compressive elastic modulus, compressive nominal stress and maximum strain of the DN and TN composite cryogels.

Sample Code	Compressive Elastic Modulus, kPa	R^2^	Compressive Nominal Stress, kPa	Strain%
DN5	40.37	0.985	31.38	52.90
TN5.1	61.57	0.995	538.20	69.02
TN5.2	42.51	0.991	48.07	55.63
DN20.1	98.01	0.998	370.49	76.30
DN20.2	126.26	0.997	14.45	13.30
DN20.3	115.16	0.999	24.75	31.18
TN20.1	163.99	0.984	316.52	68.37
TN20.2	35.15	0.999	282.35	76.15
TN20.3	99.23	0.999	11.36	21.91

**Table 3:** Isotherm parameters for the sorption of Cr_2_O_7_^2−^ anions onto SN5 cryogels and derived composite sorbents.

Isotherm Parameters	SN5	DN5	TN5.1	TN5.2
*q_m,exp_*, mg g^−1^	312	336	410	437
*Langmuir model*: $q_{e}=\frac{q_{m}K_{L}C_{e}}{1+K_{L}C_{e}}$				
*q_m_*, mg g^−1^	358.03	359.13	391.76	429.5
*K_L_*, L mg^−1^	0.0216	0.071	1.868	1.211
*R^2^*	0.8934	0.9478	0.9579	0.9889
*χ^2^*	1630	897	1074	319
*Freundlich model*: $q_{e}=K_{F}C_{e}^{N}$				
*K_F_*, mg^1−1/n^.L^1/n^.g^−1^	50.77	91.82	181.33	196
*1/n*	0.298	0.2188	0.1441	0.1418
*R^2^*	0/7401	0.7688	0.8651	0.8831
*χ^2^*	3974	3976	3447	3392
*Sips model*: $q_{e}=\frac{q_{m}a_{S}C_{e}^{N}}{1+a_{S}C_{e}^{N}}$				
*q_m_*, mg g^−1^	306.9	332.2	413.7	439.32
*a_S_*	6.146 × 10^−5^	0.0078	1.1101	1.0661
*1/n*	2.86	2.044	0.6728	0.7863
*R^2^*	0.9914	0.9951	0.9671	0.9941
*χ^2^*	130	85	840	173

**Table 4:** Isotherm parameters for the sorption of Cr_2_O_7_^2−^ anions onto SN20 cryogels and derived composite sorbents.

Isotherm Parameters	SN20	DN20.1	DN20.2	DN20.3
*q_m,exp_*, mg g^−1^	300	322	383	364.6
*Langmuir model*: $q_{e}=\frac{q_{m}K_{L}C_{e}}{1+K_{L}C_{e}}$				
*q_m_*, mg g^−1^	352.64	356	394.63	383.4
*K_L_*, L mg^−1^	0.0177	0.0354	0.2014	0.0799
*R^2^*	0.8748	0.9548	0.9335	0.9811
*χ^2^*	1830	658	1496	376
*Freundlich model*: $q_{e}=K_{F}C_{e}^{N}$				
*K_F_*, mg^1−1/n^.L^1/n^.g^−1^	44.08	67.49	124.4	99.21
*1/n*	0.3138	0.26	0.1935	0.2206
*R^2^*	0.7209	0.7891	0.7840	0.8238
*χ^2^*	4078	3330	4857	3510
*Sips model*: $q_{e}=\frac{q_{m}a_{S}C_{e}^{N}}{1+a_{S}C_{e}^{N}}$				
*q_m_*, mg g^−1^	297.75	324.6	381.6	366.05
*a_S_*	7.515 × 10^−6^	0.0046	0.0308	0.0381
*1/n*	3.29	1.75	1.92	1.378
*R^2^*	0.9978	0.9958	0.9811	0.9901
*χ^2^*	33	67	425	198

**Table 5:** Isotherm parameters for the sorption of Cr_2_O_7_^2−^ anions onto TN composite cryogels derived from SN20.

Isotherm Parameters	TN20.1	TN20.2	TN20.3
*q_m,exp_*, mg g^−1^	379	380	426.7
*Langmuir model*: $q_{e}=\frac{q_{m}K_{L}C_{e}}{1+K_{L}C_{e}}$			
*q_m_*, mg g^−1^	377.6	376.1	405.5
*K_L_*, L mg^−1^	0.3034	0.4245	3.552
*R^2^*	0.9847	0.9871	0.9718
*χ^2^*	330	280	770
*Freundlich model*: $q_{e}=K_{F}C_{e}^{N}$			
*K_F_*, mg^1−1/n^.L^1/n^.g^−1^	134.9	143.6	199
*1/n*	0.1783	0.1689	0.1345
*R^2^*	0.8441	0.8494	0.871
*χ^2^*	3352	3266	3530
*Sips model*: $q_{e}=\frac{q_{m}a_{S}C_{e}^{N}}{1+a_{S}C_{e}^{N}}$			
*q_m_*, mg g^−1^	375.41	377.4	420
*a_S_*	0.2977	0.4256	1.947
*1/n*	1.046	0.972	0.7168
*R^2^*	0.9831	0.9857	0.9771
*χ^2^*	363	309	628

**Table 6:** Isotherm parameters for the sorption of H_2_PO_4_^−^ anions onto DN and TN composite cryogels.

Isotherm Parameters	DN20.1	DN20.2	TN20.1	TN20.2	TN5.1	TN5.2
*q_e,exp_*, mg/g	295.7	367.82	373	390.4	405.3	422
*Langmuir model*: $q_{e}=\frac{q_{m}K_{L}C_{e}}{1+K_{L}C_{e}}$						
*q_m_*, mg g^−1^	331.64	397.65	378.14	376.79	382.67	407.62
*K_L_*, L mg^−1^	0.0254	0.1028	0.1938	0.8001	1.276	2.262
*R^2^*	0.9624	0.9551	0.9713	0.9551	0.9468	0.9552
*χ^2^*	492	900	596	1024	1292	1210
*Freundlich model*: $q_{e}=K_{F}C_{e}^{N}$						
*K_F_*, mg^1−1/n^.L^1/n^.g^−1^	52.6	105	123.18	157.1	167.65	194.18
*1/n*	0.284	0.2125	0.1897	0.1581	0.153	0.137
*R^2^*	0.8116	0.8109	0.8205	0.8541	0.8699	0.8593
*χ^2^*	2462	3795	3728	3318	3159	3805
*Sips model*: $q_{e}=\frac{q_{m}a_{S}C_{e}^{N}}{1+a_{S}C_{e}^{N}}$						
*q_m_*, mg g^−1^	297.47	365.58	364.83	391.1	408.93	422.45
*a_S_*	0.00301	0.0674	0.1405	0.6786	0.8178	1.421
*1/n*	1.717	1.279	1.357	0.7688	0.6539	0.7056
*R^2^*	0.9948	0.9565	0.9756	0.9557	0.9555	0.9584
*χ^2^*	68	872	507	1006	1079	1125

## Data Availability

Not applicable.

## Supplementary Materials

The following supporting information can be downloaded at: https://www.mdpi.com/article/10.3390/polym15040885/s1, Figure S1: FTIR spectra of SN5, DN5 and TN5.2 composites (sample codes are from Table 1); Figure S2: FTIR spectra of DN20.3 and TN20.3 composite sponges (sample codes are from Table 1); Table S1: Values of the average pore diameters and pore wall thicknesses of composite cryogels; Table S2: Elemental analysis given by EDX spectra of SN5, SN20 and some DN and TN composite cryogels (sample codes are from Table 1); Table S3: Swelling ratio (SR) and equilibrium water content (EWC) of the SN, DN and TN cryogels; Table S4: Values of the compressive elastic modulus for single-network cryogels as a function of number of compression cycles; Table S5: Kinetic parameters for the adsorption of Cr_2_O_7_^2−^ anions onto multi-network composite cryogels constructed onto SN5 cryogel as the first network; Table S6: Kinetic parameters for the adsorption of Cr_2_O_7_^2−^ anions onto SN20 and DN composite cryogels having SN20 as the first network; Table S7: Kinetic parameters for the adsorption of Cr_2_O_7_^2−^ anions onto TN composite cryogels constructed onto SN20 cryogels; Table S8: Kinetic parameters for the adsorption of H_2_PO_4_^−^ anions onto multi-network composite cryogels; Table S9: Selectivity coefficients for the sorption of oxyanions in binary systems; Table S10: Comparative results for the sorption of Cr_2_O_7_^2−^ and H_2_PO_4_^−^ anions by multi-network cryogels and by different sorbents from the literature.
