# Supplementary material for: Sustainable Multi-Network Cationic Cryogels for High-Efficiency Removal of Hazardous Oxyanions from Aqueous Solutions
Source: Polymers (Basel). 2023 Feb 10;15(4):885. doi: 10.3390/polym15040885 (PMC9966014; doi:10.3390/polym15040885)
Supplement: Supplementary file 1 [file polymers-15-00885-s001.zip › polymers-2205872-supplementary.pdf]

Supplementary Materials

Sustainable Multi-Network Cationic Cryogels for High-Efficiency Removal of Hazardous Oxyanions from Aqueous Solutions

Ecaterina Stela Dragan [1,*], Doina Humelnicu [2] and Maria Valentina Dinu [1]

[1] Department of Functional Polymers, "Petru Poni" Institute of Macromolecular Chemistry, Grigore Ghica Voda Alley 41 A, 700487 Iasi, Romania; vdinu@icmpp.ro
[2] Faculty of Chemistry, Alexandru Ioan Cuza University of Iasi, Carol I Bd. 11, 700506 Iasi, Romania; doinah@uaic.ro
* Correspondence: sdragan@icmpp.ro; Tel.: +40-232217454; Fax: +40-232211299

Figure S1. FTIR spectra of SN5, DN5 and TN5.2 composites (sample codes are the same with those in Table 1).

Figure S2. FTIR spectra of DN20.3 and TN20.3composite sponges (sample codes are the same with those in Table 1).

Table S1. Values of the average pore diameters and pore wall thicknesses of composite cryogels.

Table S2. Elemental analysis given by EDX spectra of SN5, SN20 and some DN and TN composite cryogels (sample codes are from Table 1).

Table S3. Swelling ratio (SR) and equilibrium water content (EWC) of the SN, DN and TN cryogels.

Table S4. Values of the compressive elastic modulus for single network cryogels as a function of number of compression cycles.

Table S5. Kinetic parameters for the adsorption of $Cr_2O_7^{2-}$ anions onto multi-network composite cryogels constructed onto SN5 cryogel as the first network.

Table S6. Kinetic parameters for the adsorption of $Cr_2O_7^{2-}$ anions onto SN20 and DN composite cryogels having SN20 as the first network.

Table S7. Kinetic parameters for the adsorption of $Cr_2O_7^{2-}$ anions onto TN composite cryogels constructed onto SN20 cryogels.

Table S8. Kinetic parameters for the adsorption of $H_2PO_4^-$ anions onto multi-network composite cryogels.

Table S9. Selectivity coefficients for the sorption of oxyanions in binary systems.

Table S10. Comparative results for the sorption of $Cr_2O_7^{2-}$ and $H_2PO_4^-$ anions by multi-network cryogels and by different sorbents from literature.

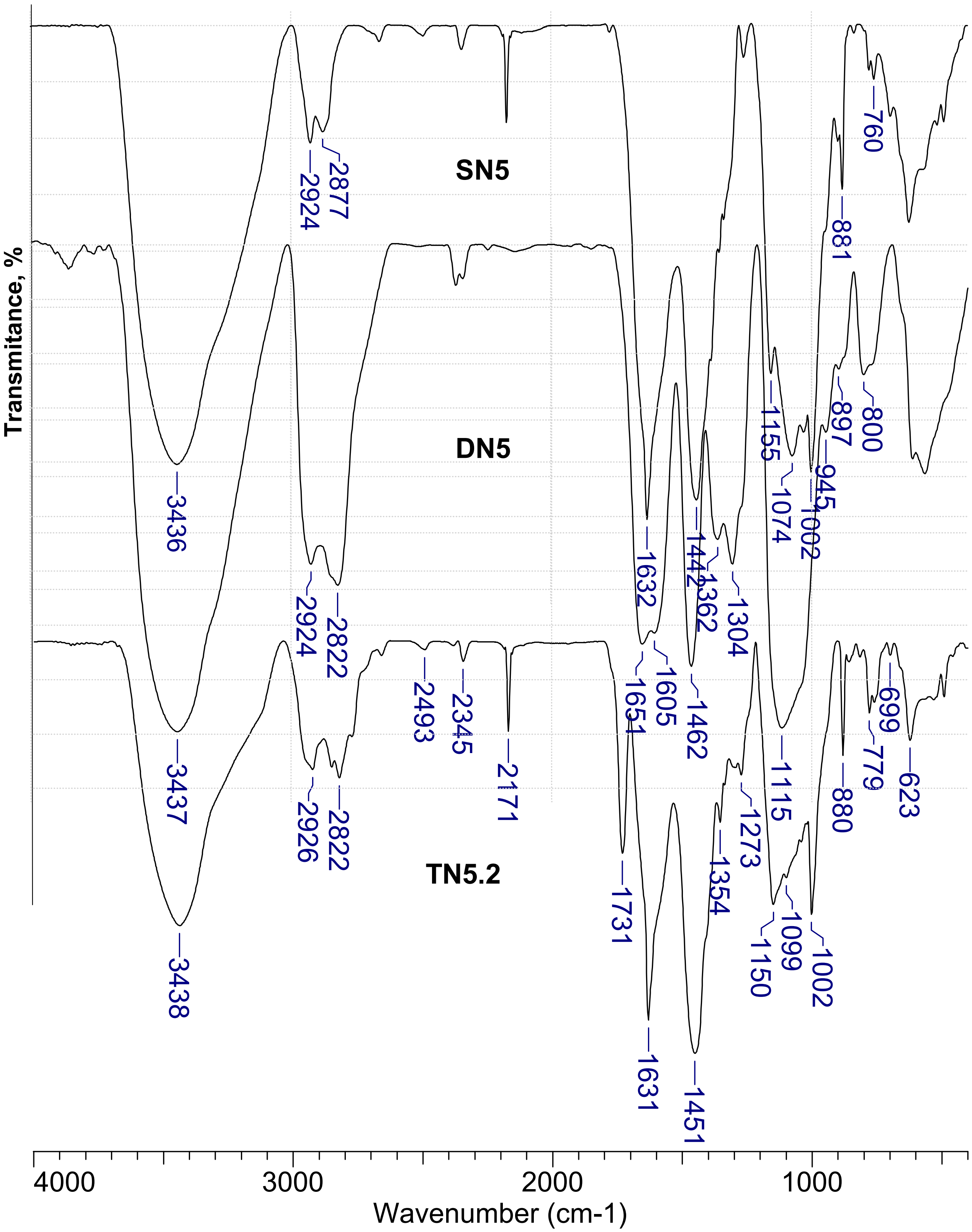

Figure S1. FTIR spectra of SN5, DN5 and TN5.2 composites (sample codes are from Table 1).

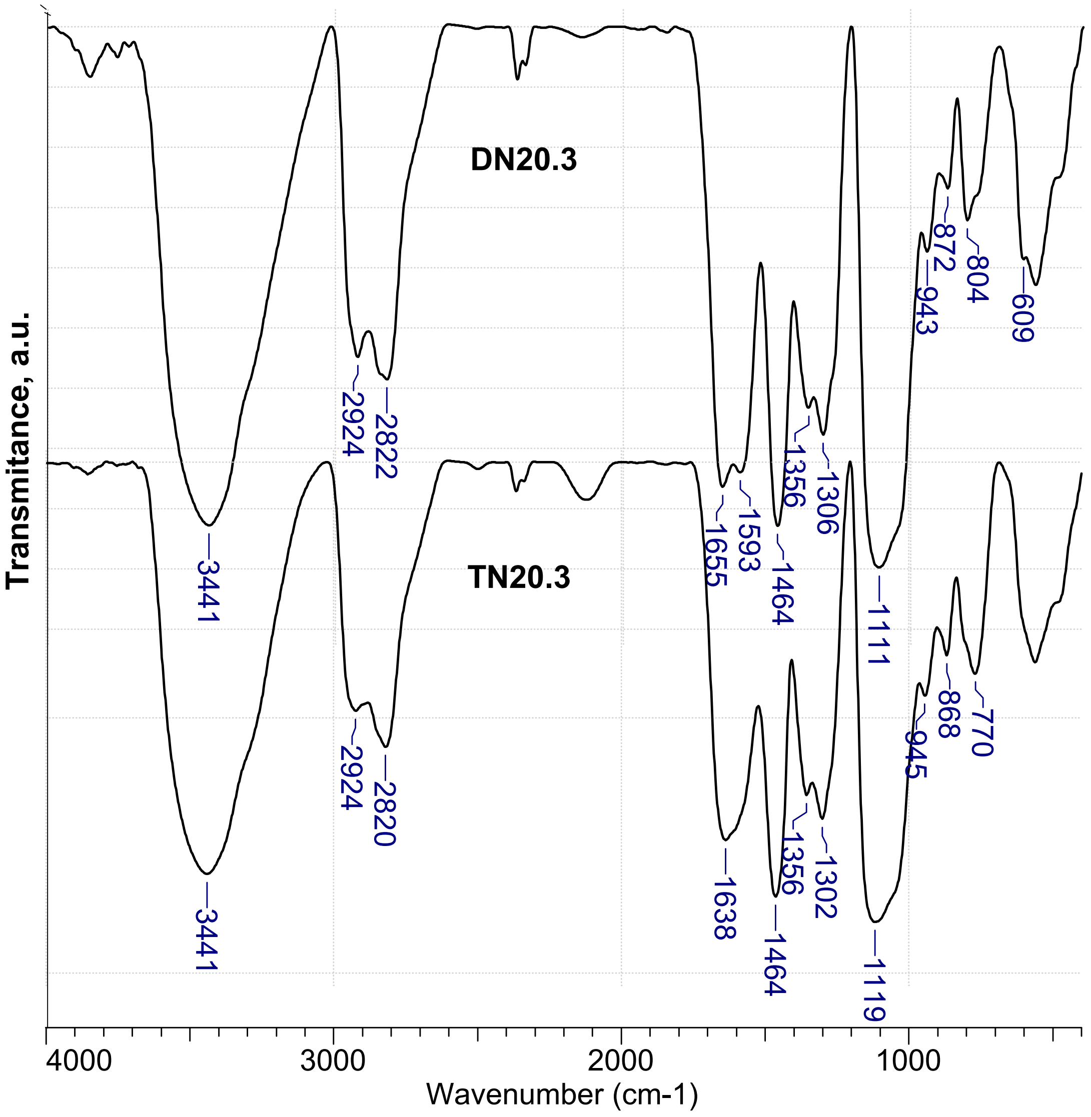

Figure S2. FTIR spectra of DN20.3 and TN20.3 composite sponges (sample codes are from Table 1).

Table S1. Values of the average pore diameters and pore wall thicknesses of composite cryogels.

Sample	Average pore diameter, µm	Pore wall thickness, µm
SN5	141.81 ± 32.56	3.50 ± 0.83
DN5	56.73 ± 12.28	10.02 ± 2.25
TN5.1	25.53 ± 4.27	18.64 ± 5.21
TN5.2	53.18 ± 6.72	20.94 ± 2.14
SN20	107.83 ± 15.47	5.73 ± 1.46
DN20.2	51.41 ± 8.71	12.95 ± 4.87
TN20.2	35.28 ± 5.44	20.76 ± 3.08

TN20.3	28.08 ± 7.09	24.77 ± 4.37

Table S2. Elemental analysis given by EDX spectra of SN5, SN20 and some DN and TN composite cryogels (sample codes are from Table 1).

Sample Code	C, wt.%	N, wt.%	O, wt.%
SN5	52.5 ± 0.15	9.5 ± 0.25	37.2 ± 0.4
DN5	63.25	12.96	23.79
TN5.1	47.46 ± 0.61	25.67 ± 2.12	26.87 ± 1.97
TN5.2	67.31	13.35	17.33
SN20	57.72 ± 3.06	7.77 ± 0.84	34.51 ± 2.29
DN20.2	67.84 ± 0.05	19.14 ± 0.51	12.96 ± 0.46
TN20.2	65.99 ± 0.5	20.45 ± 0.06	12.54 ± 0.55

Table S3. Swelling ratio (*SR*) and equilibrium water content (*EWC*) of the SN, DN and TN cryogels.

Sample Code	*SR*	*EWC*, %
SN5	64.69	98.45
DN5	9.605	89.59
TN5.1	9.48	89.33
TN5.2	9.142	89.06
SN20	45.25	97.79
DN20.1	8.52	88.95
TN20.1	6.745	85.17
DN20.2	9.17	89.87
TN20.2	8.24	87.98
DN20.3	9.011	88.903
TN20.3	8.151	87.73

Table S4. Values of the compressive elastic modulus for single network cryogels as a function of number of compression cycles.

Sample code	Number of compression cycle	Compressive elastic modulus, kPa	R^2
SN5	1	7.74	0.999
	2	4.52	0.998
	3	4.14	0.998
	4	4.17	0.998
	5	3.48	0.998
SN20	1	3.54	0.998
	2	3.52	0.997
	3	3.22	0.999
	4	2.66	0.984
	5	2.04	0.999

Table S5. Kinetic parameters for the adsorption of $Cr_2O_7^{2-}$ anions onto multi-network composite cryogels constructed onto SN5 cryogel as the first network.

Kinetic model	Parameters	SN5	DN5	TN5.1	TN5.2
$q_{e\ exp}$, mg/g		102.19	121.38	133.28	133.33
PFO	$q_{e\ calc}$, mg/g	99.63	118.56	129.4	130.99
	k_1, min^{-1}	0.0331	0.0283	0.0436	0.05767
	R^2	0.9614	0.9553	0.9591	0.9791
	χ^2	35	58	59	30
PSO	$q_{e\ calc}$, mg/g	105.83	126.3	136.6	136.6
	k_2, g mg^{-1} min^{-1}	5.213 x 10^{-4}	3.732 x 10^{-4}	5.396 x 10^{-4}	7.864 x 10^{-4}
	R^2	0.9909	0.9866	0.9927	0.9954
	χ^2	8	17	11	6.5
Elovich	α, mg g^{-1} min^{-1}	150.5	85.75	808	15076
	β, g mg^{-1}	0.086	0.0668	0.0777	0.1009
	R^2	0.945	0.9427	0.9562	0.9552
	χ^2	50	75	64	63

Table S6. Kinetic parameters for the adsorption of $Cr_2O_7^{2-}$ anions onto SN20 and DN composite cryogels having SN20 as the first network.

Kinetic model	Parameters	SN20	DN20.1	DN20.2	DN20.3
$q_{e\ exp}$, mg/g		93.41	109.34	127.59	123.76
	$q_{e\ calc}$, mg/g	91.37	106.68	124.2	120.98
	k_1, min^{-1}	0.0271	0.033	0.032	0.0295

PFO	R^2	0.9598	0.9566	0.953	0.9566
	χ^2	32	45	66	59
PSO	$q_{e\ calc}$, mg/g	97.47	113.2	132	128.72
	k_2, g mg^{-1} min^{-1}	4.594 x 10^{-4}	4.904 x 10^{-4}	4.047 x 10^{-4}	3.835 x 10^{-4}
	R^2	0.9877	0.9881	0.9878	0.9864
	χ^2	9.6	12	17	18
Elovich	α, mg g^{-1} min^{-1}	61.15	168	163.1	2542
	β, g mg^{-1}	0.0845	0.0808	0.0678	0.0972
	R^2	0.9411	0.9444	0.9474	0.9111
	χ^2	46	57	74	23

Table S7. Kinetic parameters for the adsorption of $Cr_2O_7^{2-}$ anions onto TN composite cryogels constructed onto SN20 cryogels.

Kinetic model	Parameters	TN20.1	TN20.2	TN20.3
$q_{e\ exp}$, mg/g		131.75	132.8	133.3
PFO	$q_{e\ calc}$, mg/g	128.3	128.8	130.3
	k_1, min^{-1}	0.02576	0.0382	0.0479
	R^2	0.9576	0.9579	0.9708
	χ^2	66	64	42
PSO	$q_{e\ calc}$, mg/g	137,2	137.2	136.91
	k_2, g mg^{-1} min^{-1}	3.0605 x 10^{-4}	3.791 x 10^{-4}	6.104 x 10^{-4}
	R^2	0.9891	0.9917	0.9947
	χ^2	17	13	8
Elovich	α, mg g^{-1} min^{-1}	67.64	147.67	194.3
	β, g mg^{-1}	0.0581	0.0641	0.0845
	R^2	0.9477	0.9513	0.9519
	χ^2	82	74	69

Table S8. Kinetic parameters for the adsorption of $H_2PO_4^-$ anions onto multi-network composite cryogels.

Kinetic model	Parameters	DN20.1	DN20.2	TN20.1	TN20.2	TN5.1
$q_{e\ exp}$, mg/g		103.27	127.01	128.64	123.63	128.55
	$q_{e\ calc}$, mg/g	100.49	123.88	126.76	121.86	125.97
	k_1, min^{-1}	0.0318	0.0361	0.0212	0.0221	0.0292

PFO	R^2	0.9506	0.9661	0.9757	0.9683	0.9663
	χ^2	46	47	39	46	50
PSO	$q_{e\ calc}$, mg/g	106.8	131.32	136.4	131.04	134.1
	k_2, g mg^{-1} min^{-1}	4.981 x 10^{-4}	4.596 x 10^{-4}	2.454 x 10^{-4}	2.6803 x 10^{-4}	3.618 x 10^{-4}
	R^2	0.9865	0.9932	0.9882	0.9914	0.9885
	χ^2	12	9	19	12	17
Elovich	α, mg g^{-1} min^{-1}	129.45	282	33.3	36.42	114.7
	β, g mg^{-1}	0.0836	0.0727	0.0531	0.0563	0.0639
	R^2	0.9471	0.9454	0.9314	0.9437	0.9357
	χ^2	49	75	110	83	95

Table S9. Selectivity coefficients for the sorption of oxyanions in binary systems.

Sample Code (see Table 1)	$Cr_2O_7^{2-}$			$H_2PO_4^-$			$k_{H_2PO_4^-}^{Cr_2O_7^{2-}}$
	C_e, mg/L	q_e, mg/g	$K_{dCr_2O_7^{2-}}$	C_e, mg/L	q_e, mg/g	$K_{dH_2PO_4^-}$	
SN5	26.85	97.53	3.63	30.54	92.61	3.03	1.19
TN5.2	2.14	130.48	60.97	5.72	125.71	21.98	2.77
SN20	32.18	90.42	2.81	35.09	86.54	2.47	1.14
DN20.1	21.54	1044.61	4.86	28.47	95.38	3.35	1.45
DN20.2	8.69	121.74	14.01	10.41	119.46	11.48	1.28
TN20.3	2.97	129.37	43.56	7.14	123.82	17.34	2.51

$$k_{H_2PO_4^-}^{Cr_2O_7^{2-}} = \frac{K_{dCr_2O_7^{2-}}}{K_{dH_2PO_4^-}}$$

Table S10. Comparative results for the sorption of $Cr_2O_7^{2-}$ and $H_2PO_4^-$ anions by multi-network cryogels and by different sorbents from literature.

Sorbent	Ion	pH	Temp., °C	Sorbent dose, g/L	q_m., mg/g	Ref.*
Weak base anion exchange resin impregnated with EPIDMA	$Cr_2O_7^{2-}$	4	25	0.6	194	[10]
3D-Ordered Macroporous Cross-Linked PS-*g*-PDMAEMA	$Cr_2O_7^{2-}$	4	25	0.4	400	[12]
Amidoxime sorbent	$Cr_2O_7^{2-}$	5	25	~ 0.444	130.65	[13]
Strong base anion exchanger embedded into CS/PVAm cryobeads	$Cr_2O_7^{2-}$	5.5	25	1.25	318	[14]
Magnetite modified chitosan	$Cr_2O_7^{2-}$	3	25	3	58.14	[16]

cinnamaldehyde						
TN5.2(CS/PEI18/PDMAEMA)	$Cr_2O_7^{2-}$	4	25	0.75	439	This work
Zirconium molybdate and macroporous anion exchange resin (ZMAE)	$H_2PO_4^-$	5.5	25	0.5	26.1**	[19]
Quaternary ammonium salt of cotton linter	$H_2PO_4^-$	7	30	~ 6.7	30.13	[21]
Zr-loaded magnetic CS/PVA IPN hydrogel	$H_2PO_4^-$	6.5	22 ± 1	0.25	50.76**	[22]
Zr(IV)-crosslinked carboxymethyl cellulose/carboxymethyl chitosan hydrogels	$H_2PO_4^-$	2	25	n.p.	93.5	[23]
TN20.2(CS/PEI25/PEI18)	$H_2PO_4^-$	4	23	0.75	391.1	This work
TN5.2(CS/ PEI18/PDMAEMA)	$H_2PO_4^-$	4	23	0.75	422.45	This work

*The number of references is identical with that given in the main text. **mg-P/g sorbent.
